# Optical percutaneous needle biopsy of the liver: a pilot animal and clinical study

**DOI:** 10.1038/s41598-020-71089-5

**Published:** 2020-08-26

**Authors:** Viktor Dremin, Elena Potapova, Evgeny Zherebtsov, Ksenia Kandurova, Valery Shupletsov, Alexander Alekseyev, Andrian Mamoshin, Andrey Dunaev

**Affiliations:** 1 Research & Development Center of Biomedical Photonics, Orel State University, Orel, 302026 Russia; 2 Aston Institute of Photonic Technologies, School of Engineering & Applied Science, Aston University, Birmingham, B4 7ET UK; 3 Faculty of Information Technology and Electrical Engineering, Optoelectronics and Measurement Techniques Unit, University of Oulu, Oulu, 90570 Finland; 4 Department of Anatomy, Operative Surgery and Emergency Medicine, Medical Institute, Orel State University, Orel, 302026 Russia; 5 Orel Regional Clinical Hospital, Orel, 302028 Russia

**Keywords:** Cancer, Optical spectroscopy, Surgical oncology, Biomedical engineering, Biophotonics

## Abstract

This paper presents the results of the experiments which were performed using the optical biopsy system specially developed for in vivo tissue classification during the percutaneous needle biopsy (PNB) of the liver. The proposed system includes an optical probe of small diameter acceptable for use in the PNB of the liver. The results of the feasibility studies and actual tests on laboratory mice with inoculated hepatocellular carcinoma and in clinical conditions on patients with liver tumors are presented and discussed. Monte Carlo simulations were carried out to assess the diagnostic volume and to trace the sensing depth. Fluorescence and diffuse reflectance spectroscopy measurements were used to monitor metabolic and morphological changes in tissues. The tissue oxygen saturation was evaluated using a recently developed approach to neural network fitting of diffuse reflectance spectra. The Support Vector Machine Classification was applied to identify intact liver and tumor tissues. Analysis of the obtained results shows the high sensitivity and specificity of the proposed multimodal method. This approach allows to obtain information before the tissue sample is taken, which makes it possible to significantly reduce the number of false-negative biopsies.

## Introduction

The incidence of malignant tumors of the abdominal cavity has increased rapidly in recent decades. Liver cancer is the sixth most commonly diagnosed cancer and the fourth leading cause of cancer death in the world. Primary liver cancer includes hepatocellular carcinoma (in 75–85% of cases) and intrahepatic cholangiocarcinoma (in 10–15% of cases), as well as other rare types^1^. The liver is a common site for metastasis originated from the lung, pancreas, kidney, breast, and stomach.

Despite the high level of mortality in the later stages of tumor progression, the cure rate can be high if a disease is detected at an early stage. Modern cancer diagnosis requires histological and cytological analysis of tumors, and therefore liver percutaneous needle biopsy (PNB) remains the gold standard for liver diseases diagnosis^2^. PNB includes a core biopsy (CB) with a minimum needle inner diameter of 1.0 mm and a fine-needle aspiration biopsy (FNAB) with a diameter of less than 1.0 mm^3^. The undeniable advantages of fine-needle biopsies are minimal injury to liver tissue and a low risk of complications. CB is a more traumatic procedure, but nevertheless it demonstrates high diagnostic potential because it produces high-quality histology samples of larger size^4^. Such biological samples provide more diagnostic information on oncology disorders (the presence of invasion, histological type of tumor, etc.), which is of primary importance in determining the treatment strategy in each clinical case and plays a critical role in the transition to personalized medicine. In this study, we consider Chiba-type biopsy needles with a beveled edge, known as aspirating needles. These needles are widely utilized to obtain a sample suitable for cytological examination. However, the application of a proper rotatory technique allows one to take tissue fragments for histological analysis directly from these needles^5^. At the same time, an adequate morphological assessment of focal formations and diffuse changes in the liver is possible when using needles of 16–18G^2,5^. This approach allows to obtain both cytological and histological material, and a large needle diameter reduces the risk of complications due to fewer passes.

Although the PNB procedure have medical benefits, the probability of false-negative results may reach 10%^6^. This can be attributed to the presence of nondiagnostic samples, the heterogeneous structure of tumors, and, more rarely, to incorrect histopathological assessment (diagnostic error). A sampling error is associated with the problems related to needle tip visualization, physiological movements in organs, and the patient’s involuntary movements, which causes the tissue to shift out of the area defined for the biopsy. Due to the heterogeneity of cancer cells, the samples may involve tissues showing fat necrosis, desmoplasia, or inflammatory cells that can occur between cancer cells^7^. Thus, the development of new methods for improving the efficiency of the standard PNB procedure still remains an issue of primary significance in terms of adequate diagnosis and treatment of cancer.

Optical technologies hold exceptional potential for targeting the right areas that are most sensitive to changes in the molecular and morphological structure of biological tissues. Besides, these techniques provide real-time information on tissue status. So, this approach can be used to reduce the number of undiagnosed samples, increase the sensitivity of PNB, and improve the accuracy and safety of biopsy manipulations. This approach is most adequate for diagnosis of a suspect tissue in a short time without interruption of the operation, which will increase the effectiveness of cancerous tumor surgery.

The method of fluorescence spectroscopy (FS) is currently used to monitor cell and tissue metabolism^8,9^. The FS application in oncology is based on studying the differences in the intensity of spectral composition and fluorescence of healthy and malignant tissues under laser radiation in the UV or visible range of the spectrum^10^. Fluorophores, which play a role in transformations that occur in the neoplastic process, are amino acids, tryptophan and tyrosine, structural proteins, collagen and elastin, co-enzymes, NADH and FAD, and porphyrins^11,12^. NADH and FAD are important participants in the energy metabolism of cells, and therefore monitoring of their fluorescence can detect violations in cellular metabolism. These endogenous fluorophores in cells and tissues can serve as biomarkers for studying internal differences between normal and cancer tissues^13–16^. Another technique, known as diffuse reflectance spectroscopy (DRS), yields information on light absorption and scattering in tissues. The amount of absorption depends on the tissue composition, and that of scattering is related to the morphological structure of the tissue. Quantitative analysis of the reflected light detected after light-tissue interaction reveals specific morphological, biochemical, and functional features helpful in identifying tissues and seems to be promising for tissue recognition in oncology^17–19^. DRS is used separately or in combination with FS. Using this method, one can find distinctions between malignant and benign tumors, and to detect neoplastic tissues that experience significant architectural changes at the cellular and intracellular levels. The combination of several complementary optical modalities can offer more valuable information for diagnosis and treatment, which has been demonstrated in previous studies on lung cancer^20^, breast cancer^21,22^, glioblastomas^23^, and pancreatic adenocarcinoma^24,25^.

It is common to use DRS and FS techniques for tissue recognition during small diameter optical needle biopsy. To discriminate benign and malignant lesions of both superficial and inner organs, a minimally invasive medical device, Probea, with an optical fibre integrated into a 25G needle was developed based on FS. The Probea has been used efficiently for detecting tumor tissues in lung and breast cancer^26–28^. A. Keller et al. published the research on creating an optical biopsy needle to detect liver tumors by DRS^29^. F. Braun et al. developed a multispectral needle probe combined with a virtual photometric setup for *in vivo* detection of Lewis lung carcinoma^30^. The research team led by Prof. T. Ruers has done much work on studying the characteristics of *ex vivo* tissues and *in vivo* in real-time mode during a biopsy to differentiate healthy and tumorous lung, liver and breast tissue with a specially designed optical biopsy needle using DRS^31–36^ and of dual-modality DRS-FS^37–39^.

In this study, we have developed a multimodal optical biopsy setup and investigated its diagnostic capabilities by performing PNB of the liver. The application of machine learning methods made it possible to find a solution to the classification problem and to evaluate the sensitivity and specificity of the proposed method. We present the optical probe of small diameter, acceptable for use in PNB of the liver and describe the testing needle in both animal and clinical studies. In clinical conditions, we used a routine biopsy technology with Chiba-type needle under ultrasound guidance in the absence of radiation exposure.

## Results and discussion

### Optical percutaneous needle biopsy system

**Figure 1:**
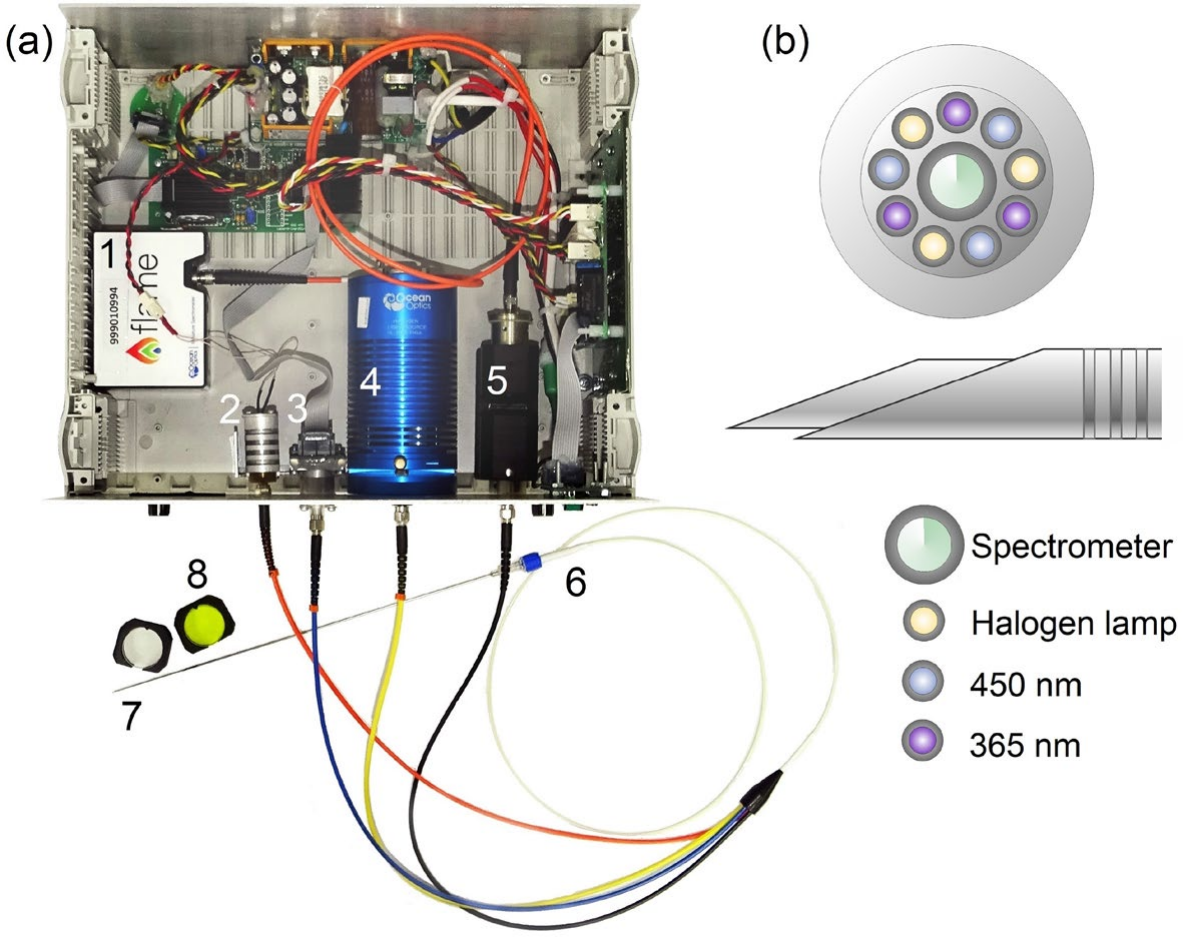
(**a**) Overview of the experimental setup: 1—spectrometer, 2—LED 365 nm, 3—laser diode 450 nm, 4—halogen source, 5—collimator and filter holder, 6—optical needle probe, 7—17.5G biopsy needle, 8—optical filters. (**b**) Arrangement of optical fibers in an optical needle probe and location of the optical probe in a standard 17.5G needle. The images of the spectrometer and halogen lamp are reproduced with permission from Ocean Insight.

A spectroscopy system with fiber optical probe containing emitting and collecting fibers was used to make fluorescence intensity measurements at 365 and 450 nm excitation and reflectance measurements in the range 400–900 nm (see Fig. 1a). The choice of wavelengths of FS channel is caused by NADH and FAD metabolic cofactors and collagen fluorescence excitation. For safety reasons, as well as to reduce the photobleaching effect, the radiation power of the 365 nm excitation source did not exceed 1.5 mW. To calculate the safe level of radiation, we used the standards of the ICNIRP^40^. The output power for the 450 nm excitation source was 3.5 mW. In the DRS channel, we used a HL-2000-FHSA tungsten halogen lamp (Ocean Insight, USA). Fluorescence and diffuse reflected radiation from tissue in the range of 400–900 nm were analyzed using a Flame spectrometer (Ocean Insight, USA). FGL400 and FGL495 filters (Thorlabs, Inc., USA) were used to attenuate the backscattered radiation of the excitation sources.

The system control and data processing operations were performed by the custom software developed in the MATLAB program environment.

The optical probe has been developed to be compatible with the 17.5G Chiba-type biopsy needle standard and has the diameter of 1 mm. The probe has 10 optical fibers (see Fig. 1b). The nine transmitting ones (100 μm each) include three fibers connected to a halogen light source, three fibers connected to a 365 nm LED, and three fibers connected to a 450 nm laser diode. The fibers are located around the central one (200 μm), which delivers the collected light to a spectrometer. The number and orientation of optical fibers inside the fiber-optic probe provide uniform and bright illumination of the diagnostic volume and allow reaching a high signal-to-noise ratio. The probe has a bevel angle of 20 degrees, which ensures a reliable contact of the probe with dense tissues. The numerical aperture of the fibers is 0.22. The optical probe is fixed in a standard biopsy needle with a special lock in the handle. The efficiency of the system has been verified in Ref.^41^. The results of the previous study have demonstrated the ability of the probe to register the changes in fluorescence caused by the oxidative phosphorylation changes in biological tissues.

Before each surgical intervention, biopsy needles were disinfected, and the experimental setup was calibrated. Control of the output power of the radiation sources was checked, a calibration spectrum was measured on a flat Spectralon diffuse reflectance standard (Ocean Insight, USA), and a dark signal was recorded to correct diffuse reflectance and fluorescence spectra.

### Assessment of the diagnostic depth

Proper evaluation of the fluorescence intensity and diffuse reflectance measurements in healthy tissues and areas of pathology is possible only if the penetration depth of radiation and the diagnostic or sampling volume are known.

**Figure 2:**
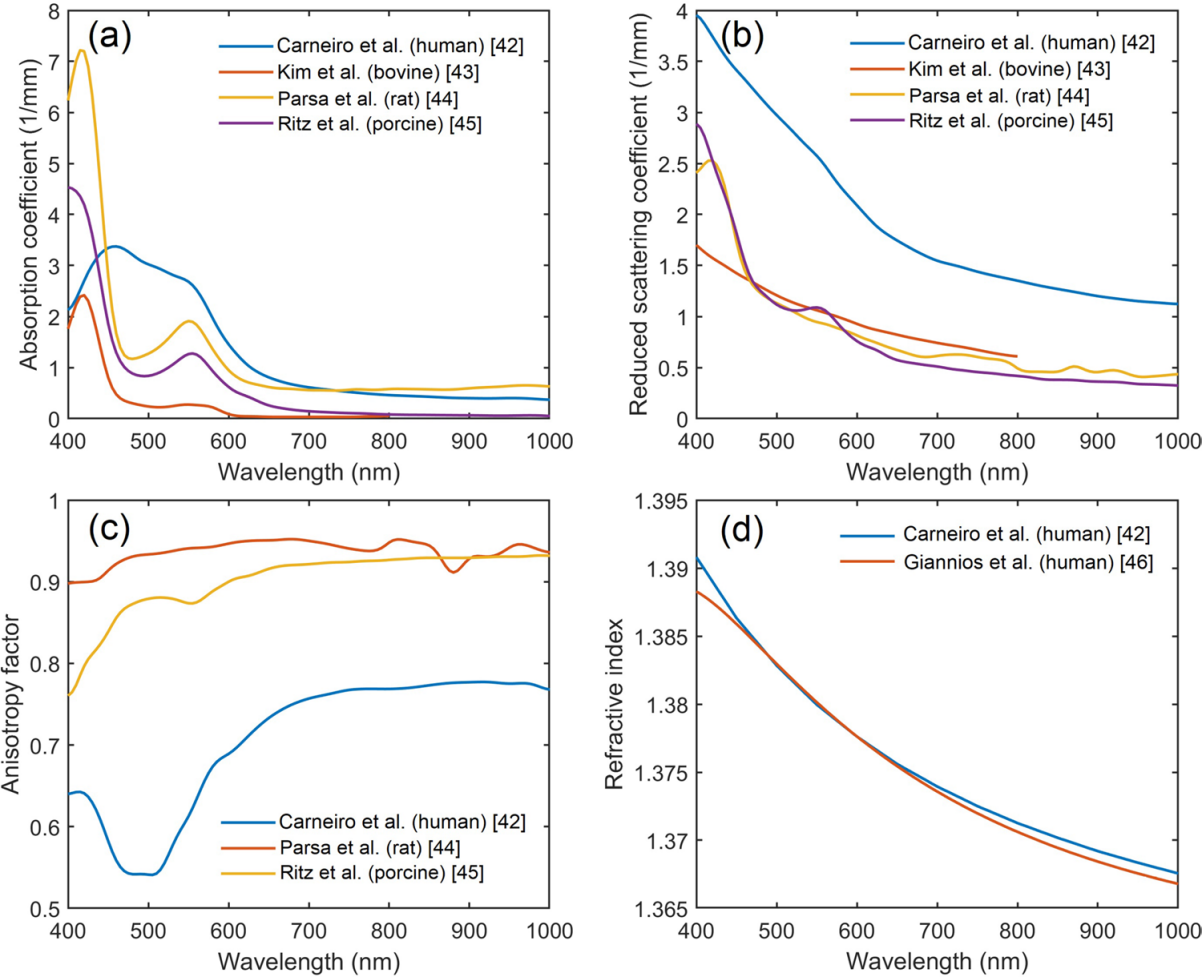
(**a**) Absorption coefficients of liver tissue, (**b**) reduced scattering coefficients, (**c**) scattering anisotropy factor, (**d**) refractive index. The optical properties were derived from a number of sources shown in the graphs and described in the text.

Figure 2a–d illustrates the optical properties of the liver in the range from 400 to 1000 nm. The optical properties of the liver were taken into account in Monte Carlo (MC) simulation of the sampling volume following the optical parameters published earlier^42–46^.

For both diffuse reflectance and the fluorescence measurements, the diagnostic volume was obtained by evaluating the probe radiation distribution using the MC method. For fluorescence measurements, the validity of this approach is supported by the fact that, according to the rule of Stokes shift, the fluorescence spectrum is shifted into the long-wave region^47^. It was also shown that, in most biological tissues, light absorption (for wavelengths of visible light spectrum) reduces with increasing wavelength. Thus, to get a lower bound estimate of the sampling volume of the measurements, it would be sufficient to assess the sampling volume of radiation for a wavelength of fluorescence excitation^48^.

**Figure 3:**
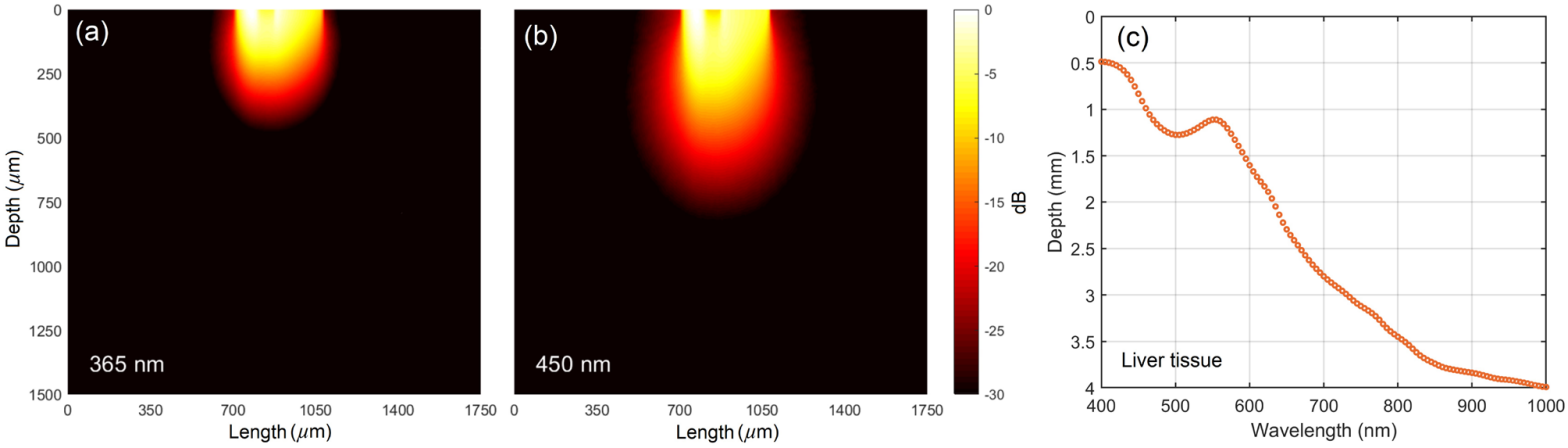
The result of modeling the sampling volume for fluorescence measurements at the excitation wavelength of (**a**) 365 and (**b**) 450 nm. (**c**) Light penetration depth in the range from 400 to 1000 nm at $10^{-3}$ of the incident light intensity for the parameters of the used ONP: distance between the fibers—220 μm, transmitting fiber diameter—100 μm, receiving fiber diameter—200 μm, numerical aperture: 0.22.

In our quantitative assessment, we consider a cross-section of the sampling volume through the centers of the source and detector positions. The sampling volume is estimated up to $10^{-3}$ of the detected intensity of light, which corresponds to the dynamic range of the commercial detectors^49^. The sampling volume is calculated on the basis of $10^9$ detected photons. The results of MC simulation of the sampling volume for fluorescence measurements at the excitation wavelength of 365 and 450 nm and light penetration depth in the range from 400 to 1000 nm are presented in Fig. 3.

As you can see from Fig. 3 for 365 and 450 nm the sensing depth does not exceed 700 μm, whereas for the NIR spectral range it reaches 3.5–4 mm. Note that the sampling volume is strongly influenced by a specific source-detector configuration and separation^50^, which can be used to develop probes with different depth sensitivity.

### Animal study

Studies in laboratory mice were conducted to preliminary evaluate the capabilities of the developed optical probe to register fluorescence and diffuse reflectance signals with a high signal-to-noise ratio from malignant and intact liver samples.

The experimental model was a hybrid BDF (C57Bl6xDBA) mouse. The animal study was approved by the local Ethics committee of Orel State University (record of the meeting No. 12 of 06.09.2018) in accordance with GLP principles. Six mice with inoculated tumors of the liver were provided by the N.N. Blokhin Russian Cancer Research Center (Moscow, Russia). The procedure of inoculation of tumors is developed and certified by this research center. The cells of low-grade hepatocellular carcinoma H33^51^ (100 μl, 50,000 cells) were inoculated into the right medial lobe of the liver of each mouse during abdominal surgery. The mice were anesthetized with Zoletil 100 (Vibrac, France) 160–190 μl. The surgical procedure included fixing the animal in the back position, preparing for the opening of the abdominal cavity, longitudinal pararectal laparotomy, and opening the abdominal cavity in the liver area. A suspension of cells was implanted by injecting through a syringe into the liver. The studies were conducted 2 months after injection in accordance with the recommendations provided about sufficient development of the tumor.

The classic PNB procedure was not performed in animal studies due to the complexity of conducting an experiment with visual control of the needle position in the body of a small laboratory animal. The procedure of laparotomy was used. During the study, mice were anesthetized with Zoletil 100, respectively, at standard dosages. Each animal was fixed on a special platform in the position on the back. Then a laparotomy, *in vivo* experimental measurements by the optical needle probe (ONP) and standard biopsy sampling procedure of liver tumor were performed. A series of optical measurements (20 fluorescence spectra for each excitation source, 100 diffuse reflectance spectra) were carried out in several areas of intact liver and tumor.

**Figure 4:**
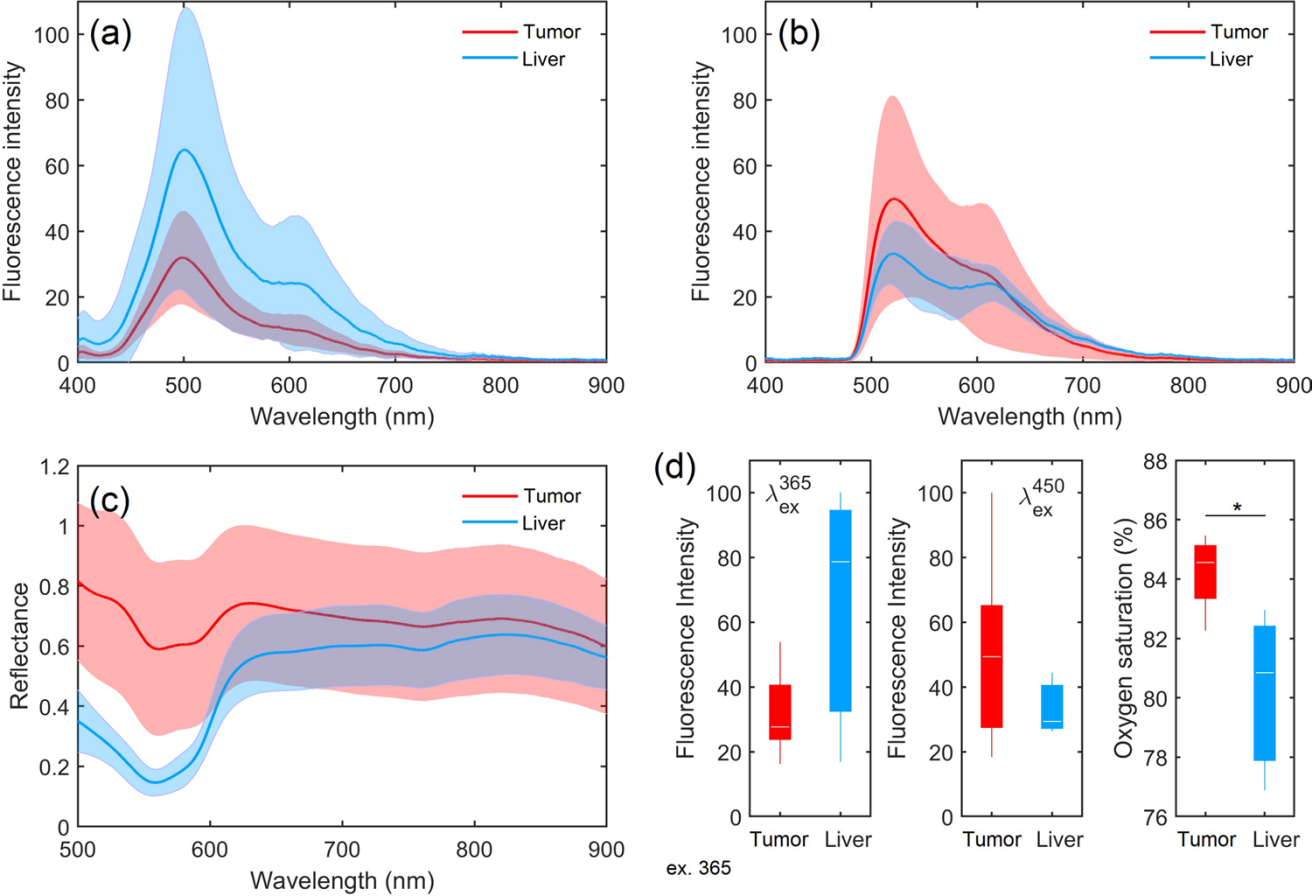
Averaged fluorescence spectra obtained from six mice under excitation wavelengths (**a**) 365 nm and (**b**) 450 nm. (**c**) Averaged diffuse reflectance spectra. (**d**) Comparison of parameters between tumor (red bars) and intact (blue bars) tissues. In each box, the central line is the median of the group, and the edges are the 25th and 75th percentiles. *Confirmed statistically significant differences (p < 0.01).

The results of averaging the fluorescence spectra are shown in Fig. 4a,b. Initial data analysis has revealed that the main feature of the collected data is a high variability of fluorescence intensities. The study of the amplitudes of endogenous fluorescence spectra of biological tissues, including the liver, is a complex task. The liver fluorescence spectrum is a superposition of many fluorophores (NADH, FAD, collagen, bilirubin, porphyrin, lipofuscin, etc.), and it also depends on the presence of various absorbers, primarily, hemoglobin and bile.

Many studies have attempted to perform the curve-fitting analysis of fluorescence spectra^52,53^. Some researchers studied the area under the curve of the normalized fluorescence spectrum^54^. However, if there is a strong blood absorption, then these approaches can lead to an erroneous result due to the presence of strong dips in the absorption spectrum of hemoglobin and reduce the area under the spectrum curve. At the same time, this is not associated with the pathological processes taking place in the probed tissue. Blood absorption can be partially compensated in the tissues with low blood volume fraction by multimodal integration of computational techniques. However, the flow rate of blood through the liver is high, and the strong effects of absorption by blood and tissues on fluorescent radiation cannot be reliably eliminated.

It follows from Fig. 4c that there are significant differences between the shapes of the diffuse reflectance spectra of normal and tumor tissues, notably in the wavelength range 600–800 nm. The tumor tissue is characterized by the oxygen saturation value, which was determined using the neural network fitting of the diffuse reflectance spectrum (see Methods). Statistically significant differences between the two types of tissue (Fig. 4d) are also visible. The oxygen saturation of tumor tissues is higher than that of intact liver tissues. One possible explanation for this situation is that the hepatocellular carcinomas and metastases from other organs are almost exclusively vascularized by the hepatic artery, whereas the liver, which does not contain tumor, has a dual vascularization (of 75 to 80% of the total volume of hepatic blood comes from the portal vein, and of 20 to 25%—from the hepatic artery)^55,56^. This can explain the increased average values of tissue saturation in tumor tissues.

The optical measurements acquired in the experiment with the tumor in a murine model permit quick assessment of the ability of the approach proposed here to collect *in vivo* spectrally resolved diffuse reflectance and fluorescence spectra. On the basis of these results, it can be concluded that an optical needle biopsy is a promising tool for detection of tumor cells in vivo.

### Clinical study

At this stage, preliminary measurements were made on the basis of the developed methodology and using the equipment at the department of interventional radiology of Orel Regional Clinical Hospital (Orel, Russia). The study was approved by the Ethics committee of Orel State University (record of the meeting No. 14 of 24.01.2019) and carried out in accordance with the 2013 Declaration of Helsinki by the World Medical Association. PNB in patients suspected of having liver malignancy was performed on a scheduled basis. The lesions had to be safely accessible. Patients at increased risk of bleeding were excluded. After receiving the description of the protocol, the patients signed informed consent indicating their voluntary willingness to participate in the study. The experiments were conducted under established protocols. The measurements were performed during the standard PNB procedure in 20 patients with supposed liver cancer. The procedure was carried after proper skin disinfection and injection of a local anesthetic (2% lidocaine hydrochloride solution). At each selected site (liver or tumor), the surgeon inserted a 17.5G Chiba-type needle (1.3 mm diameter) into the liver tissue, removed the stylet from the needle, and inserted the fiber-optic probe. The total measurement time was less than 5 min. The studies were followed by the standard procedure of biopsy sampling and histological examination. The tumor tissue samples were fixed with 10% neutral-buffered formalin, dehydrated, and embedded in paraffin. Approximately 5-μm-thick sections were stained by the haematoxylin and eosin method according to standard procedures. The resulting tissue slices were examined by light microscopy by an experienced pathologist. The main characteristics of the patients are summarized in Table 1.

**Table 1:** Differentiation of liver tumor in 20 subjects.

Subject	Gender	Age	Tumor size, mm	Histopathology	Diagnosis
1	Male	45	109 × 127	HCC, PDA	Hepatocellular carcinoma
2	Female	64	100 × 85	MDA	Metastasis gallbladder cancer
3	Female	69	26 × 24	PDA	Metastasis colon cancer
4	Female	74	30 × 30	PDA	Metastasis. Unknown source
5	Male	60	41 × 38	IM	Metastasis kidney cancer
6	Female	67	42 × 43	PDA	Metastasis caecum cancer
7	Male	68	94 × 62	MDA	Metastasis colon cancer
8	Male	58	67 × 65	IM	Metastasis pancreatic cancer
9	Female	77	40 × 45	PDA	Metastasis pancreatic cancer
10	Female	67	74 × 73	CCC, MDA	Cholangiocellular carcinoma
11	Female	78	38 × 45	SRCC	Metastasis stomach cancer
12	Female	70	47 × 50	MDA	Metastasis pancreatic cancer
13	Male	60	51 × 52	SCC	Metastasis small bowel carcinoid
14	Male	58	14 × 17	MDA	Metastasis pancreatic cancer
15	Male	66	46 × 43	SqCC	Metastasis of unknown origin
16	Male	77	46 × 38	SCC	Metastasis pancreatic cancer
17	Male	74	28 × 30	PDA	Metastasis caecum cancer
18	Male	54	45 × 38	SCC	Metastasis small bowel carcinoid
19	Male	73	50 × 40	IM	Metastasis pancreatic cancer
20	Female	54	80 × 90	SqCC	Metastasis cervical cancer

*HCC* Hepatocellular carcinoma, *PDA* Poorly differentiated adenocarcinoma, *MDA* Moderately differentiated adenocarcinoma, *CCC* Cholangiocellular carcinoma, *SRCC* Signet ring cell carcinoma, *SCC* Small-cell carcinoma, *SqCC* Squamous cell carcinoma, *IM* Insufficient material.

Of the 20 patients, the median age was 66 years (range 45 to 78 years). Ten participants (50%) were male and ten (50%) female. The results of the conventional PNB and histopathological examination of the targeted tissue revealed 20 malignancies, of which 18 were classified as metastasis from different organs. The diagnosis was made with histopathology, cytology, MRI/CT imaging, and the serum AFP tumor markers.

**Figure 5:**
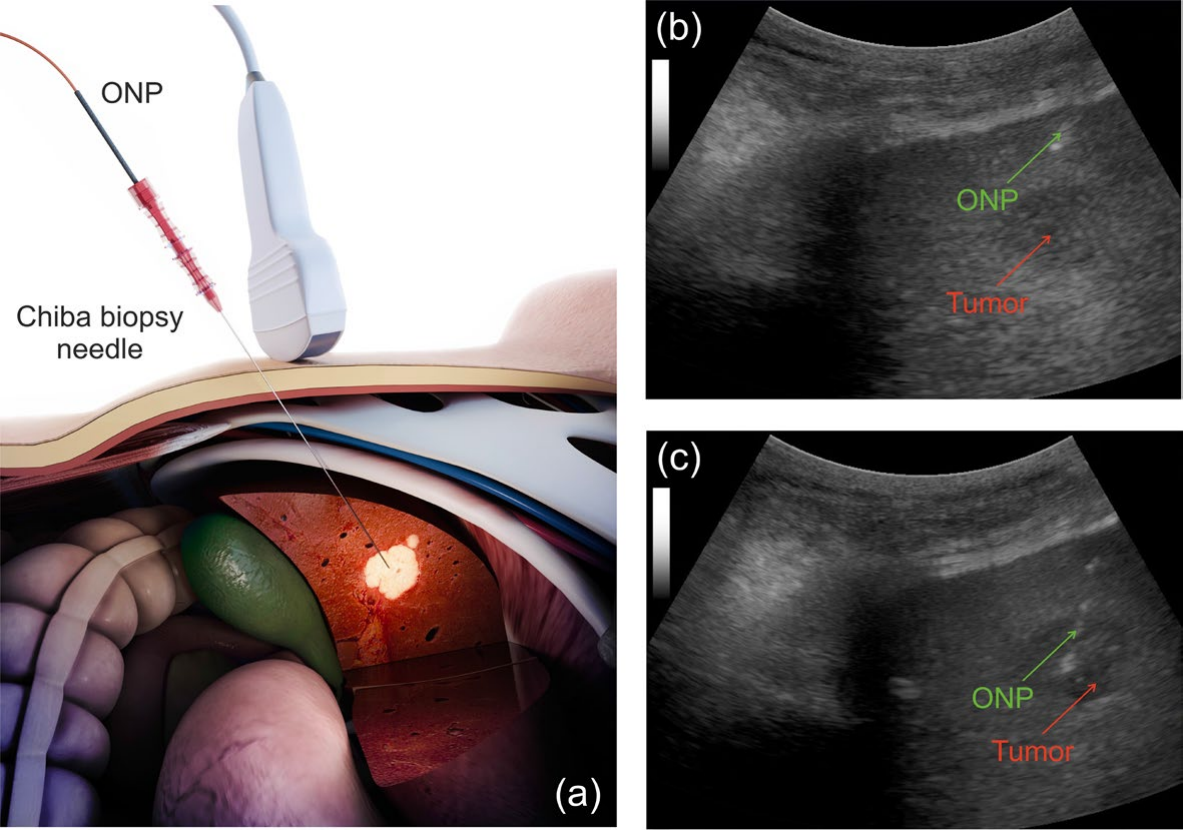
(**a**) The scheme of DRS-FS measurements during the standard PNB procedure of the liver. Positioning of the ONP (green arrow) based on ultrasound guidance in subject 14: (**b**) liver tissue and (**c**) target tumor (red arrow).

Figure 5 shows how the tissue characterization was performed using the ONP. The surgeon identified the liver lesion of interest by ultrasound examination according to the standard protocol. The ONP was inserted to reach the target point, and the measurements of fluorescence and diffuse reflectance of intact liver tissues were performed along the needle tract. 20 fluorescent spectra and 100 diffuse reflectance spectra were registered. After that, the biopsy of the target lesion was performed using the same needle.

**Figure 6:**
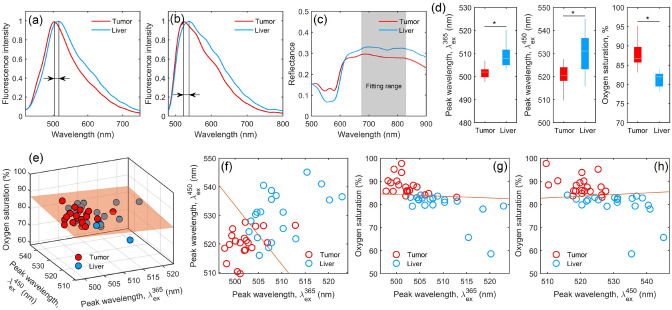
(**a**) Typical fluorescence spectra of cancer and liver tissue at 365 nm excitation, (**b**) fluorescence spectra of cancer and liver tissue at 450 nm excitation, and (**c**) diffuse reflectance spectra of cancer and liver tissue. Fitting wavelength range between 675 and 825 nm is shaded. (**d**) Comparison of parameters between cancer (blue bars) and liver (red bars) tissue. In each box, the central line is the median of the group, and the edges are the 25th and 75th percentiles. Significant differences ($p<0.01$) are confirmed statistically. (**e**) The 3D scatter plot and decision surface for the three-dimensional SVM classifier. Scatter plot and decision line (**f**) for the diagnostic parameters of peak wavelength $\lambda _{\text {ex}}^{365}$ and peak wavelength $\lambda _{\text {ex}}^{450}$; (**g**) for the diagnostic parameters of peak wavelength $\lambda _{\text {ex}}^{365}$ and oxygen saturation; (**h**) for the diagnostic parameters of peak wavelength $\lambda _{\text {ex}}^{450}$ and oxygen saturation.

We have already noted that the analysis of the fluorescence spectra amplitudes is a complicated task. However, we noticed that the liver spectra experience a red shift in wavelength, and the fluorescence peaks of tumor differ from the peaks of intact liver tissue at both excitation wavelengths (see Fig. 6a,b). The study^57^ shows that healthy tissue has more than five times more bile than tumors. A healthy liver tissue is mainly composed of hepatocytes, which ensures an even distribution of bile throughout the liver^58^. In a tumor, this structure is lost. In the case of metastases, a tumor consists of abnormal cells of the proliferating tissue embedded in the stroma. Therefore, bile is almost not expected in tumor cells of metastatic origin. In this, bile bilirubin fluorescence can shift the total spectrum of liver fluorescence to the right^52^. This parameter can serve as a qualitative diagnostic parameter for differentiating the type of tissue.

As in the animal studies, a statistically significant difference in the oxygen saturation parameter was detected between the two types of tissues. The oxygen saturation of tumor tissues was found to be higher than that of intact liver tissues. Due to the uncontrolled oncogene-driven proliferation of cancer cells, hypoxia occurs in tumors in the absence of an efficient vascular bed. With the rapid proliferation of cancer cells, the tumor exhausts nutrient and oxygen supplies from the normal vascular system and becomes hypoxic^59^. Under hypoxia conditions, there is an increase in the production of angiogenic factors and active vascularization of the tumor^60^. It is widely recognized that metastases and tumors in the liver are predominantly supplied by arterial blood^61,62^. However, a single model of tumor hypoxia is unable to describe all metabolic changes that support cell growth. There is significant intertumoral and intratumoral variability in the degree of hypoxia, in which tumor oxygenation is quite heterogeneous, and the areas of mild-hypoxia are observed, leading to severe hypoxia and necrosis, as well as areas of acute hypoxia and re-oxygenation^59^. We still face many difficulties in obtaining reliable measurements of tumor oxygen status and tumor vascularity. The efficiency of different non-invasive imaging techniques designed for monitoring the oxygenation status cannot be supported by applying direct measurement methods. Our observations demonstrate that, although the liver cancer is highly hypoxic, a significant fraction of its cells are well oxygenated.

It was shown that the diffuse reflection spectra of the liver and tumor tissues have low values in the visible range of the spectrum, which is associated with the high absorption properties of blood. In this case, the definition of saturation in the NIR region, which is less affected by the blood content, is considered the most appropriate one (see Fig. 6c).

The analyzed parameters satisfy the principles of statistical independence and a statistically significant difference between their values, calculated for the patients’ and control groups (Fig. 6d). These parameters were chosen for the synthesis of the decision rule (Fig. 6e–h). Table 2 summarizes the sensitivity and specificity parameters in different combinations. The table shows that the lowest level of error is obtained with the combination of parameters of peak wavelength $\lambda _{\text {ex}}^{450}$ and oxygen saturation or in the case of all three parameters. In general, the combination of fluorescence and diffuse reflectance measurements provides high sensitivity and specificity values, but the use of all three parameters may be excessive. The presence of two radiation sources (365 and 450 nm) can be promising in other implementations of the optical needle, e.g., in measurements of the lifetime of fluorescence. This will avoid problems with the analysis of the absolute amplitude of the fluorescence spectra and will help to perform a detailed analysis of metabolic processes in the tumor tissues of the liver.

**Table 2:** Sensitivity and specificity for different classification rules.

Parameter	1 classifier (Fig. 6e)	2 classifier (Fig. 6f)	3 classifier (Fig. 6g)	4 classifier (Fig. 6h)
Sensitivity	0.90	0.75	0.90	0.90
Specificity	0.95	0.85	0.90	0.95

The developed experimental system is designed for clinical studies and has proven to be compatible with the existing clinical workflow of percutaneous liver biopsy procedures. The conducted studies show the possibility of the combined use of FS and DRS methods in minimally invasive surgery of the liver. Simultaneous registration of fluorescence and diffuse reflectance spectra provides information on the metabolic processes in tissues and tissue morphological composition, which is of great interest in clinical practice. Biopsy remains a valuable diagnostic method, and therefore the ability to perform analyses on a real-time basis determines the relevance of optical biopsy research. The approach proposed can be used to obtain information before the tissue sample is taken, which reduces the number of false-negative biopsies.

The development of minimally invasive technologies generates a demand for diagnostic methods able to provide information *in vivo* with accuracy comparable to that of histological data. The need for such smart surgical instruments is increasing as the standard of care in liver surgery tries to spare as much healthy liver tissue as possible, which increases the number of local and segmental resections. The implementation of new approaches will increase the quality of diagnosis of biological tissue state and nature (healthy, inflamed, malignant, etc.) and, consequently, will improve the quality of treatment. Multimodal optical techniques seem rather promising and can be used as the instrumental methods for controlling tissues and mucous membranes in abdominal organs.

## Methods

### Monte Carlo simulation

Previously, we have presented the GPU-accelerated MC distributed online computational platform that implements an object-oriented concept^63,64^. This tool was used for a routine simulation of detector depth sensitivity (known also as sampling volume or diagnostic volume^64^). Briefly, the MC simulation consists of a sequential generation of trajectories of so-called photon packets from the site of photons’ entrance into the medium (“source”) to the area where the photon leaves the medium (“detector”). This approach allows the representation of photon packets and tissues structural components as objects and defines their mutual interactions. Thus, the object (photon packet) propagates through a turbid tissue-like scattering medium, represented as a set of objects, such as cells, blood vessels, etc, and interacts with them. The object-based representation of the turbid medium makes it possible to develop a realistic model of various biological tissues presenting 3D spatial variations of structural malformations associated with a particular disease^64^.

### Spectrum fitting

Oxygen saturation was measured using the previously developed approach^65,66^, which combines the artificial neural network (ANN) fitting and the training set of modelled diffuse reflectance spectra obtained through MC simulations. In short, for the training of ANN and estimation of blood oxygen saturation, the diffuse reflectance spectra of the liver were simulated by the MC technique. The GPU accelerated MC model of photon migration in scattering tissue-like media^63^ was used for routine simulation of the diffuse reflectance spectra. Matlab R2019b Deep Learning Toolbox was chosen to build and train a neural network for the retrieval of oxygen saturation level. This parameter was retrieved for the spectral range of 675–825 nm (see Fig. 6c). The optical properties of the liver described above were taken into account in modeling the diffuse reflection spectra.

### Statistical analyses

Taking into account the relatively small sample sizes, nonparametric methods were chosen to confirm the reliability of differences in the results, namely, the Mann–Whitney *U*-test. The data were inferred as statistically significant if $p<0.01$. To identify the intact liver and tumor tissue, we have applied the MATLAB Support Vector Machine Classification (SVM). SVM is an effective tool for classification problems and pattern recognition. The basic idea is to construct a hyperplane. In this study, the linear SVM was used. Lave-one-out cross-validation made it possible to ensure the stability of the classifier and to avoid over-fitting. To score the efficiency of the model constructed based on SVM, the sensitivity and specificity parameters were evaluated.
